# How effective and safe is medical cannabis as a treatment of mental disorders? A systematic review

**DOI:** 10.1007/s00406-019-00984-4

**Published:** 2019-01-31

**Authors:** Eva Hoch, Dominik Niemann, Rupert von Keller, Miriam Schneider, Chris M. Friemel, Ulrich W. Preuss, Alkomiet Hasan, Oliver Pogarell

**Affiliations:** 1 Cannabinoid Research and Treatment Group, Department of Psychiatry and Psychotherapy, University Hospital, LMU Munich, Nußbaumstr. 7, 80336 Munich, Germany; 2 APOPO, University of Agriculture, Martin-Luther-University, Morogoro, Tanzania; 3 Vitos Hospital Psychiatry and Psychotherapy, Department of Psychiatry, Psychotherapy and Psychosomatics, Martin-Luther-University, Halle-Wittenberg, Germany

**Keywords:** Mental disorders, Cannabis, Cannabinoids, THC, CBD, Medical cannabis, Treatment

## Abstract

We conducted a review of systematic reviews (SRs) and randomized-controlled trials (RCTs) to analyze efficacy and safety of cannabis-based medication in patients with mental disorders. Five data bases were systematically searched (2006—August 2018); 4 SRs (of 11 RCTs) and 14 RCTs (1629 participants) were included. Diagnoses were: dementia, cannabis and opioid dependence, psychoses/schizophrenia, general social anxiety, posttraumatic stress disorder, anorexia nervosa, attention-deficit hyperactivity disorder, and Tourette`s disorder. Outcome variables were too heterogeneous to conduct a  meta-analysis. A narrative synthesis method was applied. The study quality was assessed using the risk-of-bias tool and SIGN-checklists. THC- and CBD-based medicines, given as adjunct to pharmaco- and psychotherapy, were associated with improvements of several symptoms of mental disorders, but not with remission. Side effects occurred, but severe adverse effects were mentioned in single cases only. In order to provide reliable treatment recommendations, more and larger RCTs with follow-up assessments, consistent outcome measures and active comparisons are needed.

## Introduction

Mental disorders are among the leading causes of health impairments [25, 77, 89, 91] involving significant changes in thinking, perception, emotion, behavior and relationships [4]. They are considered as strongly restricting conditions, leading to distress for both patients and their families [19]. The etiology of mental disorders is complex, including genetic, neurobiological, psychological and environmental factors across the lifespan [64].

Recently, the efficacy and safety of cannabis-based medicines for treatment or alleviation of mental disorders has been tested more systematically. Cannabis is a flowering plant with different species producing major compounds such as the psychoactive component delta-9-tetrahydrocannabinol (THC) and cannabidiol (CBD), which have partially antagonistic effects [9, 52, 69]. THC can change mood, sensation, perception, tension, appetite, and pain; CBD has shown anxiolytic, antipsychotic, neuroprotective, anti-inflammatory and antiemetic properties [8, 34, 54, 57]. The medical use of herbal cannabinoids declined early in the twentieth century due to emerging evidence of their health risks and addictive potential [22]. However, growing interest in the substance as medicine was renewed in the 1990s with the discovery of cannabinoid receptors 1 and 2 (CB1 and CB2, respectively), endogenous ligands (endocannabinoids; *N*-arachidonoylethanolamine (anandamide/AEA) and 2-arachidonoylglycerol (2-AG)), and enzymes as part of an endogenous cannabinoid system (eCB) in the brain [49, 53]. The eCB is regarded as a fundamental regulatory apparatus connected with nearly every physiological and pathological aspects of mammalian biology [21]. The correct interplay between all these endocannabinoid system elements plays an important role in central nervous system (CNS) development, synaptic plasticity, motor control, memory, cognition, stress, emotional responses, reward and motivated behavior, appetite, pain, development and homeostasis [58, 68, 72]. Outside the brain, the eCB system is one of the crucial modulators of the autonomic nervous system, the immune system, the endocrine network, the gastrointestinal tract, the reproductive system, and in microcirculation [20]. Endocannabinoids are one of the most important systems controlling both excitatory and inhibitory neurotransmission, as well as neuroplasticity [72]. They serve as retrograde signaling messengers in GABAergic and glutamatergic synapses, as well as modulators of postsynaptic transmission, interacting with other neurotransmitters, including dopamine. Endocannabinoids also participate in the modulation of the hypothalamic–pituitary–adrenal (HPA) axis and regulation of stress [30, 75]. Preclinical and clinical data support the involvement of the eCB in the etiopathogenesis of mental disorders [24, 35, 44, 73]. Especially the CB1 receptor, which is the most abundant and widespread receptor throughout the mammalian brain, has become a target of interest [23, 38]. Reported findings from human brain studies are controversial [67] since different alterations in gene and/or protein expression of CB1 receptors have been shown to depend on the technical approach used or the brain region studied [35]. Although the picture is complex and not fully understood, the neuromodulatory function of the eCB System could be an interesting target for pharmacotherapeutic interventions in mental disorders [24, 26, 50, 57, 67, 70]. The synthesis of cannabinoid receptor agonists and antagonists, anandamide uptake blockers and inhibitors of endocannabinoid anandamide degradation has further opened up new treatment strategies [26, 72].

On the other hand, cannabis is the most frequently used illegal substance worldwide [92] and scientific evidence indicates that chronic exposure to cannabinoids may increase mental health risks, such as impaired cognition, depression, anxiety, psychoses and cannabis dependence in vulnerable persons [27, 32, 63, 86]. On a neurophysiological basis, chronic use of cannabinoids can impair CB1R function; create a loss of eCB-mediated synaptic plasticity in neural circuits, and cause addiction and negative affective states [68]. Based on these cannabis-related controversies, this paper is aimed at systematically screening the scientific literature of randomized-controlled trials (published between 2006 and 2018) to assess the efficacy and safety of cannabis-based medicines as a treatment of mental disorders.

## Methods

This systematic review followed guidance published by the Centre for Reviews and Dissemination and the Cochrane Collaboration [29]. The study protocol is registered with the Centre for Reviews and Dissemination at the University of York (UK): http://www.crd.york.ac.uk/prospero/DisplayPDF.php?ID=CRD42016053592. This work is part of a large cannabis expertise on potential and risks of cannabinoids [31] commissioned by the German Ministry of Health.

### Eligibility criteria

We searched for systematic reviews (SR) and randomized-controlled trials (RCTs) testing the efficacy and safety of medical cannabis (with or without additional medication and psychotherapy) for the treatment of mental disorders. Studies published in English or German language in the past decade were considered.

### Information sources

The data bases PsycINFO, Medline, PubMed, Embase, and the Cochrane Library were used. Hand searches were conducted, researchers in this field were contacted. Reference lists of included studies were screened. Search results and full-text articles were independently assessed by 2 reviewers; disagreements were resolved by consensus or referral to a third reviewer.

### Search

Based on the clinical research questions, we developed three detailed search strategies for identifying studies. Medical subject headings (“mesh-Terms”; U.S. National Library of Medicine 2017 https://www.nlm.nih.gov/mesh/) were used to build and pilot test search strings, which were finally adapted to the different data bases (Table 1).

### Study selection

**Fig. 1:**
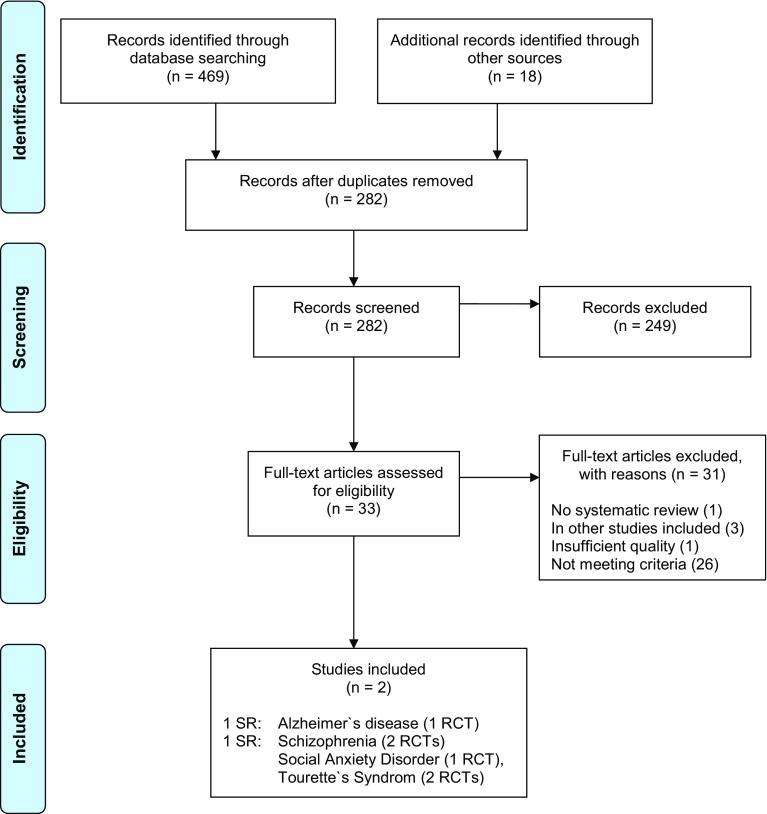
Literature search of systematic reviews (SR) (#Search 1)

**Fig. 2:**
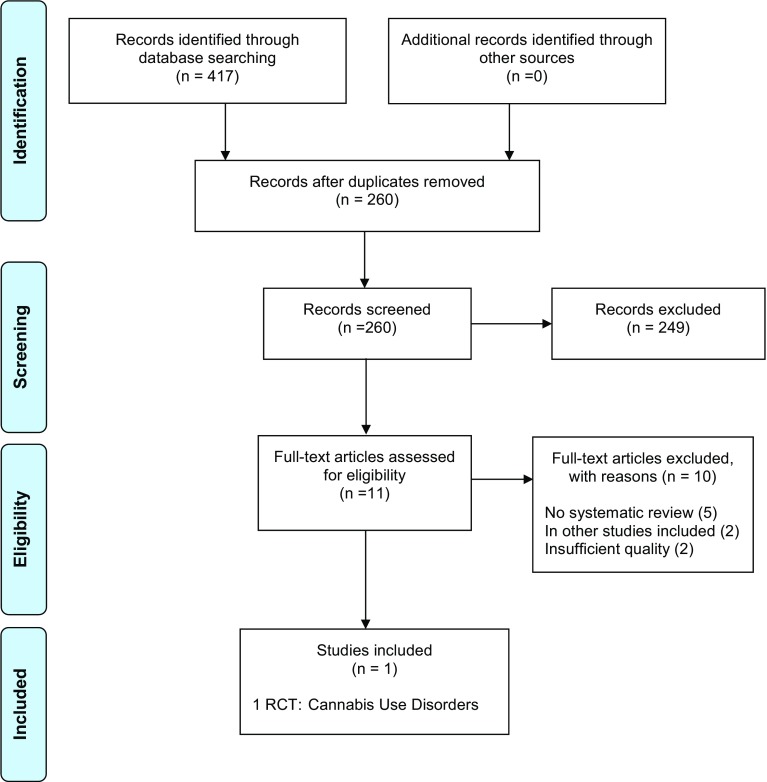
Updated search of systematic reviews (SR) and randomized-controlled trials (RCT) (#Search 2)

**Fig. 3:**
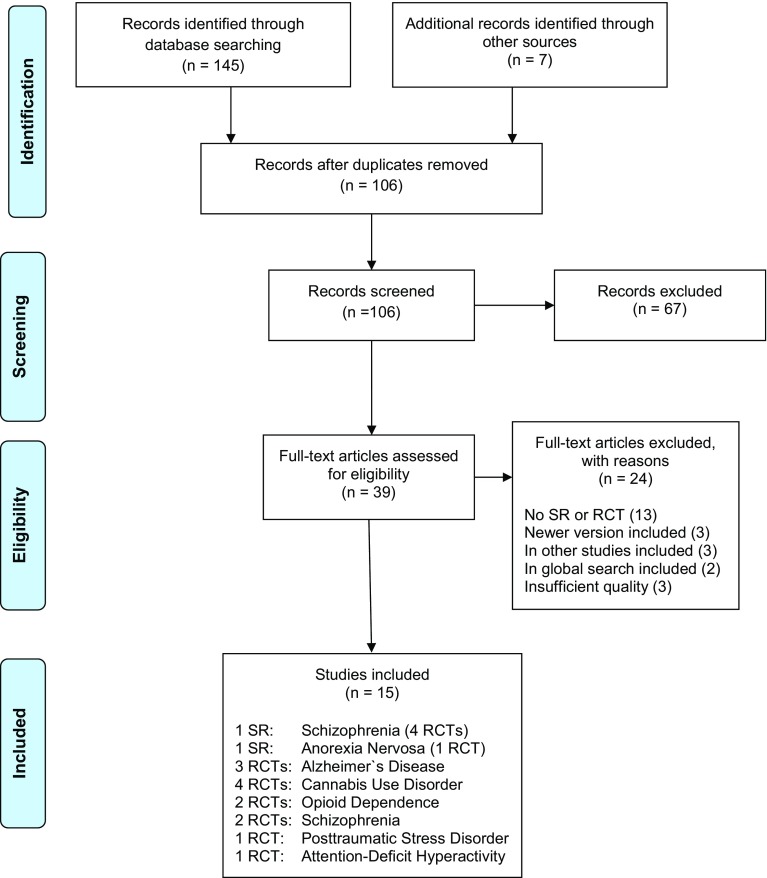
De novo research of systematic reviews (SRs) and randomized-controlled trials (RCTs)

The search process was documented in a priori defined research protocols. The study selection process (i.e., screening, eligibility, inclusion in review) was documented in PRISMA flow-charts [56] (Figs. 1, 2, 3). References are archived in EndNoteTM (EndNote X8, Clarivate Analytics).

### Data collection process and data items

Titles and abstracts of studies retrieved using the search strategies were screened independently by two researchers to identify studies which potentially meet the inclusion criteria. The full text of these potentially eligible studies was assessed for eligibility. A standardized form was used to extract data from the included studies for assessment of study quality and evidence synthesis according to the PICO scheme (i.e., baseline characteristics of patients, interventions, comparisons and outcomes). Whereever possible, the journal article was used as the primary publication because it had been peer reviewed.

### Participants

Studies were included if they reported diagnostic criteria of mental disorders [3, 4, 93].

### Interventions

Experimental condition: CB1 receptor agonists (dronabinol, nabilone, nabiximols or THC), CB1 receptor antagonists/inverse agonists (rimonabant, drinabant), cannabinoid modulators (cannabidiol) or any other cannabinoid.

Control condition: placebo and/or other medication and/or psychotherapy and/or any other intervention different from the experimental condition.

### Outcome measures

Primary outcome was the efficacy of medical cannabinoids for the treatment of any mental disorder. Secondary outcomes were tolerability and safety of medical cannabinoids.

### Risk-of-bias assessment and level of evidence

Qualitative ratings of the selected studies were conducted by using standardized protocols and tools. Systematic reviews were assessed with the Cochrane risk-of-bias tool (http://www.riskofbias.info/). RCTs were assessed for methodological quality using the SIGN-checklist [76]. Data extraction and risk-of-bias assessment were performed independently by 2 reviewers; disagreements were resolved by a third reviewer. Each study received a level of evidence, based on study type and quality [65]. All documents are available upon request.

### Synthesis of results

This systematic review applies a qualitative data synthesis approach. Due to high heterogeneity of primary outcome measures in the identified studies, no aggregated data analysis was possible. The study results were interpreted with respect to their sample size, level of evidence, risk of bias and level of heterogeneity/homogeneity.

## Results

### Study selection

The three searches resulted in 1031 screened records. 4 systematic reviews (of 11 RCTs) and 14 additional RCTs were included (PRISMA flow-charts, Figs. 1, 2, 3). The total number of study participants covered in this review is 1629. A reference list of excluded studies is available on request.

### Participants

Randomized-controlled trials assessed the following fully diagnosed mental disorders: Alzheimer`s disorder/dementia (4 RCTs), substance use disorders [opioid dependence (2 RCTs); cannabis use disorder (5 RCTs)], psychoses/schizophrenia (8 RCTs), generalized social phobia (1 RCT), posttraumatic stress disorder (1 RCT), anorexia nervosa (1 RCT), Tourette`s disorder (2 RCTs), attention-deficit hyperactivity disorder (1 RCT).

### Types of interventions and comparisons

The RCTs tested CB1 receptor agonists [dronabinol (6 RCTs), nabiximols (4 RCTs), THC (5 RCTs), nabilone (1 RCTs), CB1 receptor antagonists/inverse agonists [rimonabant (2 RCTs), drinabant (1 RCTs)] or cannabinoid modulators [cannabidiol (CBD) (6 RCTs)]. In many studies cannabis-based medicines were given as add-on to standard medication and psychotherapy.

### Results of individual studies

**Table 1:** Search process, medical subject headings (MeSH)

Search process	MeSH-terms and search setting
Search 1 (2006–2016)	Cannabis OR cannabinoid* OR hemp OR hanf (ti, ab) OR 2) Mariuana OR Marihuana OR Marijuana (ti, ab)
Search 2 (2014–2016)	Medical marihuana OR medical marijuana OR medical mariuana OR medical cannabis OR pharmaceutical marihuana OR pharmaceutical marijuana OR pharmaceutical mariuana OR pharmaceutical cannabis (ti, ab)
Search 3 (–2018)	Medical Marijuana OR Cannabinoids OR Cannabinol OR Cannabidiol OR Dronabinol (all fields), (2) Mental Disorders OR Psychotic Disorders OR Schizophrenia OR Depression OR Depressive Disorder OR Dysthymic Disorder OR Bipolar Disorder OR Anxiety Disorders OR Stress Disorders OR Post-Traumatic OR Obsessive–Compulsive Disorder OR Tourette Syndrome OR Sleep Wake Disorders OR Alzheimer Disease OR Anorexia Nervosa OR Substance-Related Disorders (all fields) (3) 1 AND 2 (4) Limit 3 to ((human AND (meta-analysis or “systematic review”)) OR (human and randomized controlled trial)) AND (English or German)

**Table 2:** Systematic reviews (*n* = 4) of RCTs (*n* = 11)* (*n* = 917 participants)

References	Study type	*N*	Patient characteristics	Intervention	Comparison	FU	Outcomes	Source of funding	Comments	LoE	ROBIS
Mucke et al. [59], Volicer et al. [85]	SR/MA	n* = 1 RCT (cross-over design) n** = 51	**Dementia-type Alzheimer** (DSM-III-R, NINCDS-ADRA Alzheimer’s Criteria)	Dronabinol (2 × 2.5 mg/d)	Placebo	n.a.	*Primary outcomes* change in body weight, caloric intake, mood changes adverse events	None	Only one RCT included in SR Small sample size Low level of evidence of RCT	1	Low
Whiting et al. [90], Leweke et al. [46]^a^, University of Cologne (2008)^a^	SR/MA	n* = 2 RCTs (1 parallel group, 1 cross-over design) n** = 71	**Schizophrenia (acute paranoid)** [DSM-IV, ≥ 36 scores in Brief Psychiatric Rating Scale]	CBD max 800 mg/day (n* = 1) (duration: 6 wks.), CBD max 600 mg/day (n* = 1) (duration: 4 wks.)	Placebo and amisulpride (max 800 mg/day)	n.a.	*Primary outcomes* Changes on Brief Psychiatric Rating Scale Changes on Positive and Negative Syndrome Scale *Secondary outcomes* Improvement of ≥ 20%, negative side effects	Swiss Federal Office of Public Health (FOPH), grant agreement 14.001443/204.0001/-1257	RCTs were judged at “high risk of bias” (GRADE) Low number of patients No meta-analysis possible Both trials allowed benzodiazepines One RCT is unpublished	1	Low
Leweke et al. [45], Meltzer et al. [55], Boggs et al. [15], Sanofi [74], Bisogno et al. [13]	SR	n* = 4 RCTs n** = 765	**Schizophrenia or psychotic disorders** (any diagnostic criteria)	Rimonabant (20 mg/d) AVE1625, cannabidiol (dosages from 600 mg/d to 800 mg/d)	Placebo (n* = 3) Amisulpride (n* = 1)	n.a.	*Primary outcomes* Brief Psychiatric Rating scale Positive and Negative Syndrome Scale	Stanley Medical Research Institute (08TGF-1257), European Commission (EU FP7 HEALTH-F2-2010-242114 - OPTiMiSE), German Federal Ministry of Education and Research (01EE1407A)	Review does not assess risk of bias or methodological quality of included studies	2	High
Whiting et al. [90], Bergama-schi [11]	SR	n* = 1 RCT n** = 24	Never-treated patients with **Generalized Social Anxiety Disorder** (SCID-CV)	CBD (single dosage of 600 mg)	Placebo (*n* = 12); healthy controls (*n* = 12)	n.a.	*Primary outcomes* Subjective ratings on the Visual Analogue Mood Scale and Negative Self-Statement scale *Secondary outcomes* Physiological measures (blood pressure, heart rate, and skin conductance), adverse effects	Swiss Federal Office of Public Health (FOPH), grant agreement 14.001443/204.0001/-1257 Swiss Federal Office of Public Health (FOPH), grant agreement 14.001443/204.0001/-1257	RCT was judged at “high risk of bias” (GRADE) Small sample size	1	Low
Whiting et al. [90], Muller-Vahl et al. [59–62]	SR	n* = 2 RCTs n** = 36	Therapy-resistant **Tourette Syndrome** **(DSM-III-R)**	Single dosage of THC (2.5 mg or 5 mg max. 10 mg/d) THC (max 10 mg/d) (duration: 6 wks.)	Placebo	n.a.	*Primary outcomes* Tourette Syndrome Symptoms List, Shapiro Tourette Syndrome Severity Scale, Yale Global Tic Severity Scale, Tourette, other assessment instruments, and adverse events	Swiss Federal Office of Public Health (FOPH), grant agreement 14.001443/204.0001/-1257	RCTs were judged at “high risk of bias” (GRADE) Small sample size in addition to a large number of scores and subscores tested Lack of statistical power Inclusion of unmedicated and medicated patients	1	Low
Hay et al. [28] Andries et al. [5, 6]	SR	n* = 1 RCT n** = 24	**Anorexia nervosa** **(DSM-IV)** for at least 5 years	Dronabinol (2 × 2.5 mg/d) (duration 4 wks.)	Placebo	n.a.	*Primary outcome* Weight *Secondary outcome* Eating-Disorder Inventory, physical activity, adverse events	None	-Intervention and placebo were added to usual care (psychotherapy and other psychotropic medication)	2	High

RCT = randomized-controlled trial, n** = number of included participants, DSM-III-TR = Diagnostic and Statistical Manual of Mental Disorders (DSM) (third edition), DSM-IV = Diagnostic and Statistical Manual of Mental Disorders (DSM) (fourth edition), DSM-5 = Diagnostic and Statistical Manual of Mental Disorders (DSM) (fifth edition), NINCDS-ADRA Alzheimer’s Criteria = criteria proposed in 1984 by the National Institute of Neurological and Communicative Disorders and Stroke and the Alzheimer’s Disease and Related Disorders Association, FU = follow-up assessment, mg/d = milligram per day, wks = weeks, n.a. = not available, CAPS = Clinician-Administered Posttraumatic Stress Disorders Scale, LoE = level of evidence (according to Oxford Centre of Evidence-based Medicine 2011) (range: 1 (“highest” – 5 (“lowest”)), SIGN = Methodology Checklist 2 of Controlled Trials: High quality (++), acceptable quality (+), low quality (−), unacceptable – reject 0

^a^Leweke et al. [46] and University of cologne [81] are included in Whiting et al. [90] and Leweke et al. [46], trials and participants are only counted once

**Table 3:** Randomized-controlled trials (*n* = 14) (*n* = 658 participants)

Reference	Study type	*N*	Patient characteristics	Intervention	Comparison	FU	Outcomes	Source of funding	Comments	LoE	SIGN
Ahmed et al. [1]	RCT (cross-over design)	n** = 10	**Dementia-type Alzheimer** (according to NINCDS-ADRA or NINCDS-AIREN Alzheimer’s Criteria)	THC (wks. 1–6, 0.75 mg; wks. 7–12, 1.5 mg) (duration: 12 wks.)	Placebo	n.a.	*Primary outcomes* Safety, pharmacodynamics and pharmacokinetics	European Regional Development Fund, Province Gelderland	Small sample size Fixed and low dosages Short intervention period Other psychotropic medication allowed	2	+
van den Elsen [82]	RCT (cross-over design)	n** = 22	**Dementia-type Alzheimer** (according to NINCDS-ADRA or NINCDS-AIREN Alzheimer’s Criteria), Neuropsychiatric Inventory score ≥ 10	THC (tablets, 2 × 0.75 mg/d in treatment blocks 1–3, 2 × 1.5 mg/d in blocks 4–6) (duration: 12 wks.)	Placebo	n.a.	*Primary outcomes* Changes on Neuropsychiatric Inventory *Secondary outcomes* Cohen-Mansfield Agitation Inventory, Zarit Burden Interview, adverse events	European Regional Development Fund, Province Gelderland	Fixed and low dosages Short intervention period Other psychotropic medication allowed	2	+
van den Elsen [83]	RCT (cross-over design)	n** = 50	**Dementia-type Alzheimer** (according to NINCDS-ADRA or NINCDS-AIREN Alzheimer’s Criteria), Neuropsychiatric Inventory score ≥ 10	THC (tablets, 3 × 1.5 mg/d) (duration: 3 wks.)	Placebo	2 wks	*Primary outcomes* Neuropsychiatric Inventory *Secondary outcomes* Cohen-Mansfield Agitation Inventory activities of daily life (Barthel Index), Quality of Life–Alzheimer’s Disease Scale, overall change, adverse events	European Regional Development Fund, Province Gelderland	Other psychotropic medication allowed Patients with severe aggressive behavior could not be included Planned number of patients not enrolled	2	+
Allsop et al. [2]	RCT	n** = 51	**Cannabis dependence** (according to DSM-IV)	Nabiximols (maximum 86.4 mg THC/80 mg CBD/d) (duration: 6 days)	Placebo	28 days	*Primary outcomes* Severity of cannabis withdrawal and cravings (Cannabis Withdrawal Scale), retention in withdrawal treatment, and adverse events. *Secondary outcomes* Post-withdrawal cannabis use, health outcomes, and psychosocial outcomes	NHMRC, GW Pharmaceuticals provided study drugs and placebo	A cognitive behavioral self-help workbook and standard detoxification care from trained nurses (including guided psychotherapy) were also provided	2	+
Levin et al. [42]	RCT	n** = 156	**Cannabis dependence** (according to DSM-IV)	Dronabinol (3 × 20 mg/d) and motivational enhancement and cognitive behavioral/relapse prevention therapy plus voucher incentives (duration: 12 wks.)	Placebo	n.a.	*Primary outcomes* Abstinence (defined as no marijuana use based on TLFB self-report) in the last two weeks of the medication phase *Secondary outcomes* Drop out of treatment, continuous abstinence, daily average amount of cannabis use, days per week of cannabis use, and per-visit withdrawal discomfort score	New York State Psychiatric Institute	Individuals who were stable and currently being treated for Axis I disorders with pharmacotherapy were not excluded from participating	2	+
Levin et al. [43]	RCT	n** = 122	**Cannabis dependence** (according to DSM-IV)	Dronabinol (3 × 20 mg/d), lofexidine (3 × 0.6 mg/d) and motivational enhancement and cognitive behavioral/relapse prevention therapy (duration: 11 wks.)	Placebo	n.a.	*Primary outcomes* Consecutive abstinence (21 days on Timeline Followback) *Secondary outcomes* Many other variables (e.g., abstinence during the last two weeks, withdrawal, craving, drop out of treatment, adverse events)	NIDA (P50DA09236, KO2 000465), lofexidine and placebo by US WorldMed	No urine screenings High drop-out rate	2	+
Trigo et al. [80]	RCT	n** = 40	**Cannabis dependence** (according to DSM-IV)	Nabiximols (max. 42 sprays/d), motivational enhancement and cognitive behavioral therapy (duration: 12 wks.)	Placebo	n.a.	*Primary outcomes* Tolerability and abstinence *Secondary outcomes* Days and amount of cannabis use, withdrawal, and craving scores	National Institutes of Health (R21DA031906) active and placebo nabiximols by GW Pharma	Participants received up to CDN$ 855 in compensation for their time	2	+
Trigo et al. [79]	RCT	n** = 16	**Cannabis dependence** (according to DSM-IV)	Nabiximols (*fixed dose*: 4 sprays/h, max. 40 sprays/d, (i.e., 108 mg THC and 100 mg CBD) *self-titrated dose*: as needed, max. 4 sprays/h, 40 sprays/d)	Placebo	n.a.	*Primary outcomes* Withdrawal, craving, medication tolerability, serious adverse events *Secondary outcomes* Many other variables (e.g., vital signs, weight, sleep, addiction severity index, brief symptom inventory, Timeline Followback for cannabis, tobacco, caffeine, alcohol)	Canadian Institutes of Health Research, active and placebo Sativex by GW Pharma	Small sample size Short duration Self-reports	3	-
Bisaga et al. [12]	RCT	n** = 60	**Opioid dependence** (DSM-IV)	Dronabinol (titrated to 30 mg/d) (duration: 8 wks.)	Placebo	3 wks	*Primary outcomes* Severity of opioid withdrawal, retention in treatment *Secondary outcomes* Hamilton Rating Scale for Depression, opiate and cannabis use, craving, adverse events	NIDA (R01 DA027124, K24 DA022412)	Study medication included also naltrexone, buprenorphine Individuals with unstable medical or psychiatric disorders were excluded	2	+
Jicha et al. [37]	RCT	n** = 12	**Opioid dependence** (adults physically dependent on short-acting opioids)	Dronabinol (up to 30 mg/session [decreased from 40 mg]) (duration: 12 wks.)	Placebo	n.a.	*Primary outcomes* Heart rate, blood pressure, pupil diameter, oxygen saturation, respiration rate, end-tidal CO_2_	NIDA (DA033932), NCATS (UL TR000117)	Unclear diagnostic assessment of opioid dependence Small sample size	2	+
Boggs et al. [16]	RCT	n** = 36	Chronic **schizophrenia** (DSM-IV)	Oral CBD (600 mg/day) (duration: 6 weeks)	Placebo	n.a.	*Primary outcomes* Verbal Short-Term Memory *Secondary outcomes* Overall Cognition as Measured on the MATRICS Consensus Cognitive Battery	Yale University	Small sample size Patients were in stable antipsychotic treatment	2	+
McGuire et al. [51]	RCT	n** = 43	Chronic **schizophrenia** (DSM-IV)	Oral CBD (600 mg/day) (duration: 6 weeks)	Placebo	n.a.	*Primary outcomes* MATRICS Consensus Cognitive Battery *Secondary outcomes* Positive and Negative Syndrome Scale	Stanley Medical Research Institute	Patients were in stable antipsychotic treatment	2	+
Jetly et al. [36]	RCT (cross-over design)	n** = 10	Canadian male military personnel with diagnosis of **posttraumatic stress disorder** (DSM-IV-TR) (and recurrent distressing dreams or staying asleep)	Nabilone tablets (max. 3.0 mg/d), other medications and psychotherapy (duration: 7 wks.)	Placebo	n.a.	*Primary outcomes* CAPS Recurrent Distressing Dreams Item *Secondary outcomes* CAPS Difficulty Falling or Staying Asleep Item, Clinical Global Impression of Change, PTSD Dream Rating Scale, General Well-Being Questionnaire	Canadian Forces Surgeon General’s Health Research Program	Very small sample size Other medications and psychotherapy Mentioned ‘modified intent-to-treat’	2	-
Cooper et al. [18]	RCT	n** = 30	**Attention-deficit hyperactive disorder** (combined type) (according to DSM-5)	Nabiximols (max. 14 spray/d) (duration: 6 wks.)	Placebo	n.a.	*Primary outcomes* Cognitive performance and activity level (head movements using the QbTest) *Secondary outcomes* ADHD and emotional lability symptoms	NIHR, NHS, Kings College, European Community, placebo and active medication by GW Pharma	Small sample size Short duration	2	+

SR = systematic review, MA = meta-analysis, Trial RCT = randomized-controlled trial, n*=number of included studies, n**=number of included participants, DSM-III-R = Diagnostic and Statistical Manual of Mental Disorders (DSM) (third edition, revised version), DSM-IV = Diagnostic and Statistical Manual of Mental Disorders (DSM) (fourth edition), DSM-5 = Diagnostic and Statistical Manual of Mental Disorders (DSM) (fifth edition), NINCDS-ADRA Alzheimer’s Criteria = Criteria proposed in 1984 by the National Institute of Neurological and Communicative Disorders and Stroke and the Alzheimer’s Disease and Related Disorders Association, SCID-CV = The Structured Clinical Interview—clinical version, THC = tetrahydrocannabinol; CBD = cannabidiol, FU = follow-up assessment, mg/d = milligram per day, wks = weeks, n.a.= not available, LoE = level of evidence (according to Oxford Centre of Evidence-based Medicine 2011) (range: 1 (“highest” – 5 (“lowest”)), ROBIS = Risk of bias assessment according to GRADE Working Group grades of evidence: (1) high quality, further research is very unlikely to change the group’s confidence in the estimate of effect; (2) moderate quality, further research is likely to have an important impact on the group’s confidence in the estimate of effect and may change the estimate; (3) low quality, further research is very likely to have an important impact on the group’s confidence in the estimate of effect and is likely to change the estimate; (4) very low quality, the group is very uncertain

*Dementia (F0 ICD-10)* 1 SR [59] of 1 RCT and 3 additional RCTs were identified [1, 82–85]. Volicer et al. [85] found positive effects on mean weight gain in both groups (dronabinol–placebo group by 3.95 kg, placebo–dronabinol group: by 3.13 kg) with a pronounced effect of dronabinol compared to placebo (*p* = 0.017) (Table 2). A reduction in negative affect (anger, anxiety and sadness) was detected during the treatment with dronabinol, which persisted during the episode following the treatment with dronabinol (ANOVA: F_(order × treatment) =_ 2.78, *df* = 1, 143, *p* = 0,12). No effects were found for caloric intake. Three patients dropped out of the study [adverse effects (*n* = 1), severe infections (*n* = 2), myocardial infarction during the placebo phase (*n* = 1)]. In a multi-centered RCT (Table 3) van den Elsen et al. [82] reported that THC did not reduce neuropsychiatric symptoms compared to placebo (block 1–3: 1.8, 97.5% CI − 2.1 to 5.8; block 4–6: − 2.8, 97.5%CI − 7.4 to 1.8). Psychiatric symptoms, agitated behavior and caregiver burden increased in both study arms during the 12 weeks trial. THC was well tolerated, as assessed by adverse event monitoring, vital signs and mobility. The incidence of adverse events was similar between treatment groups. Four non-related serious adverse events occurred. In a sample of ten patients, Ahmed et al. [1] reported that THC was rapidly absorbed and had dose-linear pharmacokinetics with considerable variation. Pharmacodynamic effects, including adverse events, were minor. Another RCT with cross-over design [84] tested the efficacy and safety of low-dose oral THC (3 × 1–5 mg/day, 3 weeks.) or placebo. Neuropsychiatric symptoms were reduced during both treatment conditions, with no difference between THC and placebo (mean difference NPI_total_: 3.2, 95% CI 23.6–10.0). No changes in scores for agitation, quality of life, or activities of daily living were found. The number of patients experiencing mild or moderate adverse events was similar (THC: *n* = 16, placebo: *n* = 14; *p* = 0.36). No effects on vital signs, weight, or episodic memory were observed. In all trials, patients could continue their standard medication. In summary, the studies provide a heterogeneous picture: superior effects (weight gain and reduction in negative affect) of THC (given in addition to standard treatment and compared to placebo) were only found in one out of three trials. Adverse effects occurred in all conditions.

*Opioid dependence (F11.20 ICD-10)* Severity of withdrawal, retention in treatment or safety were assessed in 2 RCTs (3 reports, 70 patients) [12, 37, 48] (Table 3). The first intervention [12] was dronabinol (titrated to 30 mg/day, 3 weeks) (*n* = 40) compared to placebo (*n* = 20). Patients were stabilized with buprenorphine (2 × 4 mg/day) (day 2) and received naltrexone from day 5 onwards. The study revealed a significant initial reduction in the severity of the opioid withdrawal during the initial 8-day detoxification phase for dronabinol (up to 30 mg/day) compared to placebo (*p* = 0.006). No significant difference was found in retention between the groups (35% retained in either group). The rate of successful induction onto XR-naltrexone did not differ between dronabinol (66%) and placebo (55%) groups (*χ*^2^ = 1.46, *p* = 0.23). A high number of AEs (96% vs. 91%) was reported. They were considered to be consistent with symptoms of naltrexone-related protracted withdrawal. Jicha et al. [37] and Lofwall et al. [48] tested the safety of dronabinol with *n* = 12 adults physically dependent on short-acting opioids. Participants were maintained on oral 30 mg oxycodone. The study showed that a higher dosage of dronabinol (40 mg/day) produced sustained sinus tachycardia accompanied by anxiety and panic necessitating dose reduction to 30 mg. Compared to placebo, 20 and 30 mg dronabinol produced significant increases in heart rate beginning 1 h after drug administration which lasted approximately 2 h (*p* < 0.05). Dronabinol 5 and 10 mg produced placebo-like effects. Altogether, the evidence for the usefulness of dronabinol as adjunct medication in the detoxification of opioid-dependent patients is still very small. Only one study shows a reduction in the severity of the opioid withdrawal in the initial detoxification phase. The number of reported adverse events was high in one study (naltrexone-related protracted withdrawal) while safety concerns were given in the second study for dronabinol at 20 mg and higher given in combination with oral oxycodone.

*Cannabis dependence (F12.2 ICD-10)* Craving, withdrawal and abstinence from cannabis were assessed in 5 RCTs (319 participants) in patients with cannabis dependence [2, 42, 43, 79, 80]. Trigo et al. [80] (Table 3) added nabiximols or placebo to a manualized motivational enhancement therapy and cognitive behavioral therapy. Rates of adverse events did not differ between treatment arms (*F*_1,39_= 0.205, ns). There was no significant change in abstinence rates at trial end. Cannabis use decreased in either group, without significant differences between treatment conditions (*p* = 0.179). Nabiximols reduced cannabis craving, but no significant differences between groups were observed on withdrawal scores. No serious adverse events were reported. In a previous multi-centered RCT with cross-over design, Trigo et al. [79] tested the effects of nabiximols on both craving and withdrawal symptoms among *n* = 9/16 non-treatment seeking cannabis-dependent adults. High fixed doses of nabiximols were well tolerated and, compared to placebo, significantly reduced symptoms of cannabis withdrawal during abstinence, but not craving. Self-titrated doses were lower and showed limited efficacy compared to high fixed doses. Participants reported a significantly lower “high” following nabiximols or placebo as compared to treatment as usual conditions. Nausea, diarrhea and sleep disorders were reported, but no serious AEs. Levin et al. [43] assessed abstinence (= 21 consecutive days), withdrawal, craving, and adverse events. The intervention was dronabinol (self-titrated up to 3 × 20 mg/day), lofexidine (self-titrated up to 3 × 0.6 mg/day) or placebo. In both conditions, patients received cannabis-specific manualized behavioral therapy. The trial found no treatment effect in achieving abstinence. Drop-out rates were high (48% vs. 42%). More AEs were found in the dronabinol group (e.g., dry mouth, intoxication, and hypotension), serious AEs (2 cases) were reported in the placebo lead-out phase. In a previous double-blind RCT, Levin et al. [42] tested dronabinol (2 × 20 mg/day) or placebo in addition to weekly motivational enhancement and relapse prevention therapy. There was no significant difference between treatment groups in the proportion of participants who achieved 2 weeks of abstinence at the end of the maintenance phase (dronabinol: 17.7%; placebo: 15.6%). Both groups reduced cannabis use over time (no differences between groups). Treatment retention was significantly higher at the end of the maintenance phase on dronabinol (77%), compared to placebo (61%) (*p* = 0.02). Withdrawal symptoms were significantly lower on dronabinol than placebo (*p* = 0.02). Dronabinol was well-tolerated. Allsop et al. [2] assessed the effects of nabiximols or placebo combined with standardized psychosocial interventions. Nabiximols significantly reduced the overall severity of cannabis withdrawal relative to placebo (*p* = 0.01), including effects on withdrawal-related irritability, depression, and cannabis cravings. Nabiximols patients remained longer in treatment during medication use (unadjusted hazard ratio, 3.66 [95% CI, 1.18–11.37]; *p* = 0.02), with a number needed to treat of 2.84 to achieve successful retention in treatment. The frequency (*p* = 0.59) and severity (*p* = 0.10) of adverse events did not differ significantly between groups. Both groups showed reduced cannabis use at follow-up, with no advantage of nabiximols over placebo for self-reported cannabis use (*p* = 0.75), cannabis-related problems (*p* = 0.14), or cannabis dependence (*p* = 0.89). In sum, existing trials provide a heterogeneous picture of the effectiveness of dronabinol and nabiximols in the treatment of cannabis use disorders. Four studies did not find positive effects regarding abstinence or reduction in cannabis use. Three out of five RCTs showed a significant reduction of withdrawal symptoms. Two out of three trials indicate improved craving and one study indicates improved retention in treatment. Adverse effects were more frequently reported in the intervention group in two of three studies. Future studies should use consistent outcome variables (e.g., assessment of abstinence) and treatments (manualized CBT/relapse prevention) in order to provide comparable findings.

*Psychoses/Schizophrenia (F20 ICD-10)* Two systematic reviews [45, 90] of 6 RCTs [13, 15, 46, 55, 74, 81] and two new RCTs [16, 51] were found (*n* = 887 participants). In the studies of Leweke and colleagues individuals with psychosis received CBD (800 mg/day, 4 weeks) [46] or CBD (600 mg/day, 2 weeks) [81]. The comparisons were amisulpride (max 800 mg/day) and placebo, respectively. Primary outcomes were psychiatric symptoms [66] and positive and negative symptoms [39]. Both studies found significant improvements vs. baseline on days 14 and 28 for CBD and amisulpride; no difference was found between groups. The authors reported side effects for all medications, but the side-effect profile (extrapyramidal symptoms, weight gain, prolactin values) was considered superior for CBD vs. amisulpride [46]. McGuire et al. [51] conducted a double-blind parallel-group trial where patients with chronic schizophrenia received CBD (1000 mg/day; *n* = 43) or placebo (*n* = 45) add-on to the existing antipsychotic medication. Participants were assessed before and after treatment using the Positive and Negative Syndrome Scale [39], the Global Assessment of Functioning scale (GAF) [40], Brief Assessment of Cognition in Schizophrenia (BACS) [41] and the improvement and severity scales of the clinical global impression scale [17] (CGI-I and CGI-S). After 6 weeks of treatment, compared to the placebo group, the CBD group had lower levels of positive symptoms (PANSS: treatment difference = − 1.4, 95% CI = − 2.5, − 0.2) and were more likely to have been rated as improved (CGI-I: treatment difference = − 0.5, 95% CI = − 0.8, − 0.1) and as not severely unwell (CGI-S: treatment difference = − 0.3, 95% CI = − 0.5, 0.0) by the treating clinician. Patients who received CBD also showed greater improvements which fell short of statistical significance in cognitive performance (BACS: treatment difference = 1.31, 95% CI = − 0.10, 2.72) and in overall functioning (GAF: treatment difference = 3.0, 95% CI = − 0.4, 6.4). CBD was well tolerated, while rates of adverse events were similar between the CBD and placebo groups. Boggs et al. [16] conducted a 6-week parallel-group RCT. The intervention was a fixed-dose study of oral CBD (600 mg/day) or placebo augmentation in *n* = 36 stable antipsychotic-treated patients diagnosed with chronic schizophrenia. This study compared the cognitive, symptomatic, and side effects of CBD vs. placebo in a clinical trial. No main effect of time or drug on MATRICS Consensus Cognitive Battery [10], but a significant drug × time effect was observed (*p* = 0.02). Post hoc analyses revealed that only placebo-treated subjects improved over time (*p* = 0.03). There was a significant decrease in PANSS total scores over time (*p* < 0.0001), but there was no significant drug × time interaction (*p* = 0.18). Side effects were similar between CBD and placebo, with exception being sedation, which was more prevalent in the CBD group. Overall, CBD was well tolerated with no worsening of mood, suicidality or movement side effects. Leweke et al. [45] (LoE: 2; ROBIS: high) included 3 further published double-blind RCTs (of unclear risk of bias). The interventions were Rimonabant [15, 55] and drinabant (AVE1625) [74] compared to placebo (*n* = 3). The two CB1R antagonists/inverse agonists tested in schizophrenia had no significant effects on psychopathology and cognition. Rimonabant and drinabant (AVE1625) were withdrawn from worldwide marketing due to psychiatric side effects [45]. Altogether, cannabidiol plus existing antipsychotic medication showed beneficial effects. Improvements in psychotic symptoms and cognition were not superior to antipsychotic medication or placebo in 3 studies. More studies with larger sample sizes are needed to systematically investigate these effects.

*Generalized social phobia (ICD 10 F40.11)* One systematic review [90] including 1 RCT [11]. This RCT tested the effects of one experimental session of a simulated public speaking test on treatment-naive individuals with generalized social anxiety disorder (*n* = 36). Participants received either a single dose of CBD (600 mg; *n* = 12) or placebo (*n* = 12). Both groups were compared to healthy controls (*n* = 12). Subjective ratings on the Visual Analogue Mood Scale (VAM) [33, 88] and physiological measures (blood pressure, heart rate, skin conductance) were measured at six different time points during the tests. Medication with CBD significantly reduced anxiety, cognitive impairment and discomfort in their speech performance, and significantly decreased alert in their anticipatory speech. The placebo group presented higher anxiety, cognitive impairment, discomfort and alert levels when compared with the control group. No significant differences were observed between the CBD group and healthy controls in the negative self-statement scores, cognitive impairment, discomfort, and alert factors. The increase in anxiety induced by the public speaking tests on subjects with generalized social anxiety disorders was reduced with the use of CBD, resulting in a similar response as the healthy controls. No adverse effects were found for CBD. More studies with larger sample sizes are needed to replicate the results of this study.

*Posttraumatic stress disorder (ICD 10 F43.12)* One RCT (n = 10 participants) [36] assessed recurrent distressing nightmares in a sample of Canadian male military personnel with PTSD (DSM-IV). The intervention was nabilone (0.5 mg, titrated to the effective dose (nightmare suppression) or reaching a maximum of 3.0 mg/7 weeks). Subjects were allowed to continue psychotherapy and any other medication. The mean reduction in nightmares as measured by the CAPS Recurring and Distressing Dream scores [14] were − 3.6 ± 2.4 and − 1.0 ± 2.1 in the intervention and control group (*p* = 0.03). Mean global improvement as measured by the Clinical Global Impression of Change (CGI-C) [17] was 1.9 ± 1.1 (i.e., much improved) and 3.2 ± 1.2 (i.e., minimally improved) in the intervention and control group (*p* = 0.05). Five out of 10 (50%) were much improved on nabilone vs. 1 out of 9 (11%) on placebo. For the General Well-Being Questionnaire [71] improvements were 20.8 ± 22 and − 0.4 ± 20.6 in the nabilone and placebo groups, respectively (*p* = 0.04). No improvement of sleep intensity and quality was reported. Every second participant reported adverse events (nabilone group: 50%; control group: 60%). No event was severe nor resulted in a drop-out. More studies with larger sample sizes are needed to replicate these study results.

*Anorexia nervosa (ICD 10 F50.0)* One systematic review [28] of 1 RCT [5–7] was found. This study tested the effects of dronabinol (2 × 2.5 mg/day; 4 weeks) as add-on to standard psychotherapy and eating management in a sample of women with severe (> 5 years) anorexia nervosa (DSM-IV-TR) (*n* = 25). Primary outcomes were weight, behaviors and beliefs (Eating-Disorder Inventory II) [78] as well as physical activity. The trial found weight gain in both groups, with larger effects in the active treatment group (1 kg) vs placebo (0.34 kg). No changes were found in the Eating-Disorder Inventory and in the duration of physical activity. Adverse effects were reported in both groups (no serious adverse effects). More studies with larger sample sizes are needed to replicate these results.

*Attention-deficit hyperactivity disorder (ADHD) (ICD 10 F90)* The effects of Nabiximols compared to placebo were tested in one RCT (*n* = 30 adults) [18]. Patients currently treated with stimulants were asked to stop their medication for 1 week before their baseline assessments and for the duration of the study. Patients on long-acting medications, such as atomoxetine, were excluded from the study. The primary outcome was cognitive performance and activity level; secondary outcomes included ADHD and emotional lability symptoms. For the primary outcome, no significant difference was found (ITT-analysis) (Est = − 0.17, 95% CI − 0.40 to 0.07, *p* = 0.16). For secondary outcomes, improvements in hyperactivity/impulsivity (*p* = 0.03) and a cognitive measure of inhibition (*p* = 0.05) were found, but not in inattention (*p* = 0.10) or emotional lability (*p* = 0.11). Results did not meet significance following adjustment for multiple testing. One serious (muscular seizures/spasms) and three mild adverse events occurred in the active group and one serious adverse event (cardiovascular problems) in the placebo group. More studies with larger sample sizes are needed to replicate the results of this study.

*Tourette’s disorder (ICD 10 F95)* One systematic review [90] identified 2 RCTs (3 reports, *n* = 36 Patients) [60–62] in which therapy-resistant Tourette`s disorder (*n* = 12/*n* = 24) were assessed. The interventions were THC (1 × max. 10 mg) and THC (titrated to 10.0 mg/day, 6 weeks), respectively. Comparisons were placebos (identical in taste and appearance). Multiple outcome variables were assessed (e.g., Tourette`s Syndrome Clinical Global Impressions Scale), Shapiro Tourette-Syndrome Severity Scale, Yale Global Tic Severity Scale (YGTSS) [87]. Both studies found improvement in various Tourette-related outcomes, of which not all reached statistical significance. Adverse effects were reported in both groups (no serious adverse effects). More studies with larger sample sizes are needed to replicate the results of these two studies.

### Synthesis of results

Due to a large heterogeneity of patient groups, interventions, comparisons and outcome criteria, data were not sufficient to calculate effect sizes and odds ratios.

## Discussion

Only recently, cannabis-based medicine was tested more systematically for the treatment of mental disorders. The aim of this systematic review was to analyze its efficacy, tolerability and safety in patients with a diagnosed mental disorder. The literature research identified 4 SRs (of 11 RCTs) and 14 additional RCTs. A total of 1629 patients was examined, meeting criteria of 9 classified diagnoses of mental disorders (DSM-III-R, DSM-IV, DSM-5). Across studies, methodological limitations reduced the confidence in the evidence for several reasons: (1) for most indications, only single RCTs with small sample sizes (e.g., general social anxiety disorder, attention-deficit hyperactivity disorder) have been published. (2) In cases where more studies are available, results were mixed (e.g., dementia, cannabis use disorders) or did not consistently reach statistical significance (e.g., schizophrenia). A large variety of outcome variables were used and they were not comparable across studies.

The scientific literature shows, that among all cannabis-based medicines, THC-based preparations have been tested most frequently as treatment for mental disorders: Nabiximols (4 RCTs) [2, 18, 79, 80], dronabinol (6 RCTs) [5–7, 12, 42, 43, 48, 85], THC (5 RCTs) [1, 60–62, 82, 83], and nabilone [36]. All trials were placebo-controlled; other medication (e.g., benzodiazepines) and psychotherapy were available in most studies.

The largest number of controlled studies (7 RCTs) and thus best evidence available, is for THC-based medicine (nabiximols, dronabinol) as an adjunct to other interventions in the treatment of substance use disorders (cannabis dependence, opioid dependence). In cannabis dependent patients, a reduction of cannabis withdrawal symptoms was found in 3 RCTs [2, 42, 79], but not found in 2 studies [43, 80]. Two out of three trials reported improved craving and one study indicates improved retention in treatment. Significant effects on abstinence or a reduction in substance use were not found between intervention and comparison groups. Adverse effects were more frequently reported in the intervention group in two of three studies. Future studies should use consistent outcome variables (e.g., assessment of abstinence) and treatments (manualized CBT/relapse prevention) to provide comparable findings. Among opioid dependent patients, a reduction in the severity of opioid withdrawal was reported in one study [12]. The evidence of THC-based preparations in the treatment of further mental disorders is also small, the effects are mixed. In Alzheimer`s disease results are inconsistent for the improvement of neuropsychiatric symptoms, mood and agitation in patients (3 RCTs) [82, 83, 85]. A consistent improvement of tics and behavioral problems was found in 2 studies including patients with therapy-resistant Tourette`s disorder. Not all results reached statistical significance [60–62]. For all other mental disorders, only single studies are available regarding the efficacy of THC-based medicine in these conditions. The reported therapeutic benefits in primary outcomes are: weight gain in patients with therapy-resistant anorexia nervosa (1 RCT) [5–7], improvement of nightmares and well-being in patients with posttraumatic stress disorder (1 RCT) [36]. In patients with attention-deficit hyperactivity disorder improvement in cognition and activity level did not reach statistical significance (1 RCT) [18]. Adverse events occurred in all studies, with no difference between groups. However, safety concerns appeared during opioid withdrawal for dronabinol at 20 mg and higher [12].

Cannabidiol as a treatment of mental disorders was tested in 6 RCTs. Most data is available for patients with psychoses and schizophrenia. Cannabidiol (CBD) plus existing antipsychotic medication was associated with significantly lower levels of positive symptoms compared with the placebo group in 1 RCT [51]. Three further RCTs also found improvements in both psychotic symptoms and cognition, which fell short of statistical significance if compared to antipsychotic medication [46] or placebo [16, 81]. Positive anxiolytic effects of CBD were shown in patients with generalized social anxiety compared to a placebo group and healthy controls (1 RCT) [11]. No adverse effects have been reported for cannabidiol as treatment for mental disorders.

Other cannabis-based medicines were rimonabant and drinabant, which have been tested in 3 RCTs for the treatment of schizophrenia [15, 55, 74]. These studies found no benefits on cognition and psychiatric symptoms. Due to serious adverse effects, rimonabant and drinabant were withdrawn from the market.

In summary, the evidence for efficacy and safety of cannabis-based medicines as a treatment for mental disorders is still small. Reported improvements were mostly assessed in single RCTs with small sample sizes. In order to get a clearer picture of potential therapeutic effects, to reveal differential indications (which group of patients is most likely to benefit from cannabis preparations) and to allow a generalization towards naturalistic samples of patients, more clinical research of high methodological quality is needed. The required studies should be multi-centered, randomized and controlled, including large samples sizes. The currently existing trials generally tested cannabis-based medication for a few days up to several weeks. No follow-up assessments were conducted. Long-term data, however, are essential to get information on optimal treatment duration, sustained cannabinoid effects (e.g., tolerance, symptoms of withdrawal, cognition, quality of life, level of functioning) and safety. Consistent outcome measures (e.g., disorder remission, change in symptom severity, hospitalization, patient or care person’s perception of improvement) should be used to assess the efficacy of cannabis medicine. Furthermore, to assess the potential of cannabis-based medicine in comparison to existing treatment options, future studies should have active control groups such as first-line pharmacological treatments (e.g., antidepressants, antipsychotic medication) and psychosocial treatments (e.g., manualized cognitive behavioral therapy) as comparisons.

## Conclusion

THC- and CBD-based medicines were associated with improvements of several symptoms of mental disorders, but not with remission. Side effects can occur, but severe AEs were mentioned in single cases only. The overall confidence in the evidence is low. To provide reliable treatment recommendations, more high-quality RCTs with larger sample sizes are requested.
