# Correction to: How effective and safe is medical cannabis as a treatment of mental disorders? A systematic review

**DOI:** 10.1007/s00406-019-00999-x

**Published:** 2019-04-05

**Authors:** Eva Hoch, Dominik Niemann, Rupert von Keller, Miriam Schneider, Chris M. Friemel, Ulrich W. Preuss, Alkomiet Hasan, Oliver Pogarell

**Affiliations:** 1 Cannabinoid Research and Treatment Group, Department of Psychiatry and Psychotherapy, University Hospital, LMU Munich, Nußbaumstr. 7, 80336 Munich, Germany; 2 APOPO, University of Agriculture, Martin-Luther-University, Morogoro, Tanzania; 3 Vitos Hospital Psychiatry and Psychotherapy, Department of Psychiatry, Psychotherapy and Psychosomatics, Martin-Luther-University, Halle-Wittenberg, Germany

## Correction to: European Archives of Psychiatry and Clinical Neuroscience (2019) 269:87–105 10.1007/s00406-019-00984-4

The article “How effective and safe is medical cannabis as a treatment of mental disorders? A systematic review”, written by Eva Hoch, was originally published Online First without open access. After publication in volume 269, issue 1, page 87–105 the author decided to opt for Open Choice and to make the article an Open Access publication. Therefore, the copyright of the article has been changed to © The Author(s) 2019 and the article is forthwith distributed under the terms of the Creative Commons Attribution 4.0 International License (http://creativecommons.org/licenses/by/4.0/), which permits use, duplication, adaptation, distribution and reproduction in any medium or format, as long as you give appropriate credit to the original author(s) and the source, provide a link to the Creative Commons license and indicate if changes were made.

The original article has been corrected.

